# Reproductive Strategies of *Aedes albopictus* (Diptera: Culicidae) and Implications for the Sterile Insect Technique

**DOI:** 10.1371/journal.pone.0078884

**Published:** 2013-11-13

**Authors:** Clelia F. Oliva, David Damiens, Marc J. B. Vreysen, Guy Lemperière, Jérémie Gilles

**Affiliations:** 1 Insect Pest Control Laboratory, Joint FAO/IAEA Division of Nuclear Techniques in Food, International Atomic Energy Agency, Vienna, Austria; 2 Institut de Recherche pour le Développement, MIVEGEC (IRD 224-CNRS 5290-UM1-UM2), Montpellier, France; 3 Centre de Recherche et de Veille sur les Maladies Emergentes dans l′Océan Indien, Sainte Clotilde, La Réunion, France; University of Massachusetts, United States of America

## Abstract

Male insects are expected to optimize their reproductive strategy according to the availability of sperm or other ejaculatory materials, and to the availability and reproductive status of females. Here, we investigated the reproductive strategy and sperm management of male and virgin female *Aedes albopictus*, a mosquito vector of chikungunya and dengue viruses. The dynamics of semen transfer to the female bursa inseminalis and spermathecae were observed. Double-mating experiments were conducted to study the effect of time lapsed or an oviposition event between two copulations on the likelihood of a female double-insemination and the use of sperm for egg fertilization; untreated fertile males and radio-sterilised males were used for this purpose. Multiple inseminations and therefore the possibility of sperm competition were limited to matings closely spaced in time. When two males consecutively mated the same female within a 40 min interval, in ca. 15% of the cases did both males sire progeny. When the intervals between the copulations were longer, all progeny over several gonotrophic cycles were offspring of the first male. The mating behavior of males was examined during a rapid sequence of copulations. Male *Ae. albopictus* were parceling sperm allocation over several matings; however they would also attempt to copulate with females irrespective of the available sperm supply or accessory gland secretion material. During each mating, they transferred large quantities of sperm that was not stored for egg fertilization, and they attempted to copulate with mated females with a low probability of transferring their genes to the next generation. The outcomes of this study provided in addition some essential insights with respect to the sterile insect technique (SIT) as a vector control method.

## Introduction

Biting mosquito females from the *Aedes* genus can transfer viruses and nematodes to humans, some of which are responsible for severe diseases such as dengue, yellow fever, chikungunya, and lymphatic filiariasis [1]. *Ae. aegypti* and *Ae. albopictus* are the two major species responsible for disease transmission. They are very aggressive and effective in invading and settling into new regions [2] which has resulted in increased disease transmission risk [3], [4], epitomizing the urgent need for the development and implementation of sustainable integrated vector control programs. These programs can include the sterile insect technique (SIT) (classical SIT, or SIT using *Wolbachia*-modified or genetically modified mosquitoes) where the release of sterile males can reduce wild local mosquito populations. Male mosquitoes are not directly involved in disease transmission; however, understanding their mating behavior has critical consequences for such control tactics, which rely entirely on the males' capacity to mate [5]. Current knowledge on *Aedes* reproductive physiology and behavior concerns mainly female *Ae. aegypti*. Little is known about *Ae. albopictus* mating strategies, despite the fact that it is rapidly becoming a growing threat in Europe and other parts of the world making the development of control strategies for this pest insect increasingly pertinent. This study attempts to bring a better understanding of male *Ae. albopictus* reproductive strategies and their implications for the sterile insect technique.

The reproductive strategy of male insects shapes its fitness through production and management of sperm, capacity to acquire mates, ability to compete with other males, female choice, and investment in offspring [6]–[9]. Male insects commonly invest little in individual sperm but instead increase reproductive fitness by maximizing both the number of sperm produced and the number of matings [10]. Nevertheless, sperm production can be costly and some insect species appear to have limited amounts of large sperm at emergence [11]. In addition, sperm depletion after several matings has been demonstrated in numerous insect species [12]–[16]. In male invertebrates, mature sperm is stored in seminal vesicles or in the testes before mating and is usually delivered to females either freely (as ejaculates) or in a package (as spermatophores). The resources needed to package sperm could be a limiting factor for male fitness [6], [17]–[19]. Males are thus expected to optimize the use of their sperm or ejaculates according to the availability of sperm or other ejaculatory materials and to the availability and reproductive status of females [7].

*Ae. albopictus* males can acquire mates in pair matings or through the formation of small swarms [20]. Both strategies generally occur near blood-meal sources [20], giving the male a high probability to meet either virgin females during their first blood-meal or non-virgin females seeking a blood-meal for a subsequent gonotrophic cycle (GC). In such situations the likelihood of copulating with non-virgin females is high, irrespective of whether the females had the first mating a few days or an instant ago. It is not yet known whether males can recognize female mating status and adapt their mating strategy accordingly. However, the secretions produced by the male accessory gland (MAG) and transferred into the female bursa inseminalis (BI) together with sperm [21]–[24] are thought to prevent further insemination, similarly to the mating plug in *Anopheline* species [25], [26] or to the spermatophore in other insect species [12]–[15], [27]–[29]. Moreover, the MAG products are also known to modify female behavior [6], [17]–[19], [30]–[32]. In *Ae. albopictus* and *Ae. aegypti* males, the MAG secretion transferred during insemination induces long-term sexual refractoriness in females, but this only takes into effect 2 to 3 days after insemination [31]. However, *Aedes* females have been observed to copulate several times [33], and though they are considered as primarily monoandrous, examples of multiple insemination exist both in the laboratory and in the field. In the laboratory, Gwadz and Craig [34] reported that 7.5% of female *Ae. aegypti* exposed to several males produced offspring fathered by multiple males. Under semi-field conditions, 14% of *Ae. aegypti* females were found to be carrying sperm from two males after 48 hours [35]. In addition, 26% of wild-caught *Ae. albopictus* females produced multi-sired progeny [36]. However, data seems to indicate that the likelihood of multiple inseminations in *Ae. aegypti* would be a result of either an incomplete insemination during the first mating [34] or the occurrence of the two mating events within a few hours [22].

Due to the existence of this temporary physical plug and chemical induction of female refractoriness, a high male-male competition can be assumed in *Aedes* species. Moreover, in insects, multiple inseminations could lead to sperm competition in females [37]. All these parameters are known to influence the sperm management of male insects [38], [39]; however, sperm management has not been studied yet in mosquito species. The purpose of this work was therefore to study sperm transfer to the reproductive tract of *Ae. albopictus* females and the management of sperm by conspecific males according to their own mating history and female reproductive status. The effect of different time intervals and oviposition before the second mating on the likelihood of female multiple-insemination was investigated. Untreated (fertile) and sterilized (X-ray treated) males were used in order to test the fate of sperm from two different males in twice-mated females. In addition, the assessment of the mating ability of untreated and sterilized males gives important indications for population control programs with an SIT component.

## Materials and Methods

### Rearing procedures

The colony of *Ae. albopictus* used for the experiment originated from field collections in Rimini, Northern Italy and has been maintained under laboratory conditions at the Centro Agricultura Ambiente, Bologna, Italy. The strain was transferred to the FAO/IAEA Insect Pest Control Laboratory (IPCL), Austria in 2010, where adults were kept in a climate-controlled room maintained at 27±1°C and 60±10% relative humidity with a light regime of LD 12∶12 h photoperiod, including dusk (1 h) and dawn (1 h). Adults were kept in standard 30×30×30 cm cages (Megaview Science Education Services Co, Ltd, Taiwan) and continuously supplied with 10% wt: vol sucrose solution with 0.2% methylparaben [40]. Females were offered a blood meal weekly on defibrinated bovine blood using a Hemotek feeding apparatus with modified plates [41] (Discovery Workshops, Accrington, Lancashire, United Kingdom) and were allowed to oviposit in plastic beakers containing deionized water and lined with crêpe paper (Sartorius Stedim Biotech GmbH, Göttingen, Germany). Five days after the blood meal, the egg paper was removed from the cage and left to dry at ambient conditions for 24 h. The eggs were kept in a closed container for at least one week to allow embryonic development. Eggs were hatched in a closed 1-litre jar containing 0.7 litre of deionized water, 0.25 g of Bacto Nutrient Broth® and 0.05 g of yeast. Hatched larvae (less than 4 h old) were transferred to plastic trays (40×29×8 cm) containing 0.5 litre of deionized water and fed a diet of finely ground (224 μm-sieved) Koi Floating Blend® (Aquaricare®, Victor, New York, USA). Pupae were collected and placed in small plastic cups inside a fresh adult cage for emergence.

### Male sterilisation

Male pupae were irradiated in an X-ray irradiator (RS 2400, Rad Source Technologies Inc.) containing horizontal cylindrical canisters, which rotate around an X-ray tube. Pupae were maintained with minimal water using plates in a cylindrical canister designed specifically for irradiation of mosquito pupae. Male pupae, aged 24–40 hours, were irradiated with 40 Gy, which induces ca. 99% sterility in adult males [42]. A dosimetry system was used to measure the dose received by the lot based on Gafchromic® dosimeter HD-810 film (International Specialty Products, NJ, USA); three dosimeters were included with each lot of insects and read after irradiation with a Radiachromic® reader (Far West Technology, Inc., California, USA). After irradiation, males were allowed to emerge in a laboratory cage and were provided with sugar solution. Hereafter, fertile and irradiated males are referred to as untreated and sterilized males, respectively.

### Pair mating procedure and parameters measured

For all of the following experiments, pair matings were carried out in an emergence tube (diameter 11.4 cm, height 9.7 cm, BioQuip, Rancho Dominguez, CA). One male and one female were introduced into the tube and the tube was gently shaken from time to time to stimulate encounter and copulation. Two observers simultaneously took part in all treatments in order to minimise any observer effect. The latency period before copulation and the copulation duration were recorded with a stopwatch, with the insertion of the *aedeagus* being considered the start of copulation. Immediately after copulation either both male and female were removed and isolated in a small tube (diameter 2 cm, height 10 cm) or one of the mate was offered a successive opportunity to copulate with a new partner, depending on the type of experiment. In the text, females referred to as “mated” or “twice-mated” are females who performed one or two copulations of normal length, whether or not the copulation (s) resulted in insemination. When semen was actually transferred as determined by female dissection or egg hatch, females were referred to as “once-inseminated” or “twice-inseminated”.

Males and females used in the experiments were 2 days old, except when otherwise specified; all mosquitoes used in these experiments originated from the main colony with larvae reared under standard conditions. Temperature was kept constant at 27±1°C across all experiments.

### Experiment 1. Copulation of virgin males and insemination success

The relationship between copulation and effective insemination was determined using virgin females in 105 couples with untreated males and 88 couples with sterilized males. Each individual was only used for one mating. Copulation duration was recorded. Females were killed by freezing not less than one hour after mating to allow sufficient time for sperm to reach the spermathecae. The spermathecae and BI were then dissected in a drop of saline solution. The number of inseminated spermathecal capsules was recorded as well as the presence of seminal fluid in the BI. Digital photos of well-preserved female left wings (or right where left wings were damaged) were taken and used to carry out measurements that can be related to the body size using ‘analySIS B’ software (Olympus Soft Imaging Solutions, Germany). Wings were measured from the distal edge of the alula to the end of the radius vein (excluding fringe scales).

### Experiment 2. Sperm transfer dynamics in the female reproductive tract

To determine the structural changes in BI contents following insemination and the dynamics of sperm transfer from the BI to the spermathecae, pairs consisting of a virgin female and either an untreated or sterilized male were allowed to mate. After copulation, the female was killed by exposure to ether vapors and the reproductive tract was dissected to observe transfer of sperm from the BI to the spermathecae and the appearance of the semen inside the BI. Mosquitoes were dissected 1–6 min (n = 84), 15–30 min (n = 15), 40 min-1 h (n = 21), 6 h (n = 18), 24 h (n = 20) or 48 h (n = 34) after the start of copulation (half of the males dissected at each time period were untreated and half were sterilized).

### Experiment 3. Transfer of semen in once- or twice-mated females

**Figure 1:**
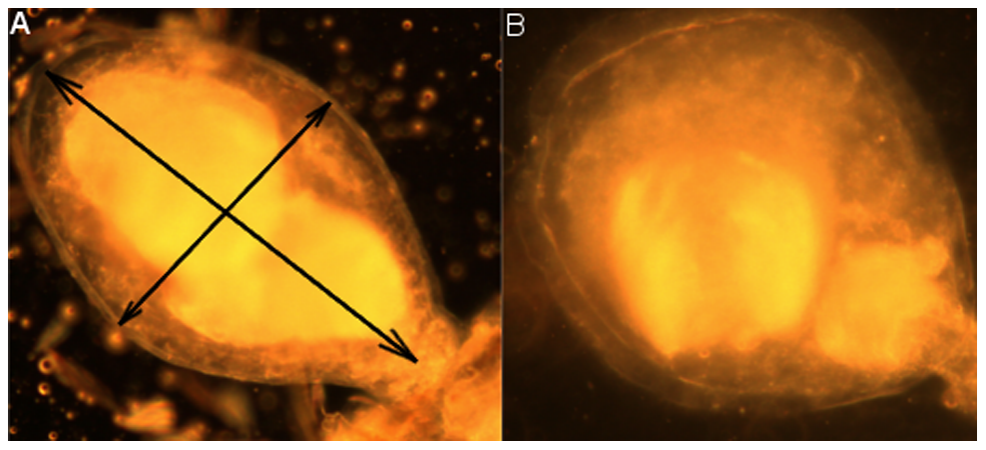
Photographs of the bursa inseminalis (BI) of female *Ae. albopictus* inseminated twice as shown by the two distinct masses of semen. The arrows in A indicate the measurements taken for the bursa surface analysis.

To determine if a female that copulated twice possessed more semen in the BI and in the spermathecae compared to once-mated females, the BI surface was measured and the spermathecae were dissected. Females were mated with either one untreated male (n = 61), one sterilized male (n = 15); or two untreated males (n = 66). Only uninterrupted first copulations that lasted more than 30 seconds (decided based on the results from experiment 1) were kept for the double mating group. A second male was introduced immediately after removal of the first; in all cases the two mating opportunities occurred within a 1-hour interval. Females were dissected one hour after the copulation; the presence or absence of seminal fluids in the BI and the number of inseminated spermathecal capsules were recorded, and two pictures of the BI were taken at magnification 100 and 200 X. In order to minimize sample-handling bias, the same operator carried out all dissections and took all photographs. The relative surface area of the female BI was used as an indicator to estimate differences in the amount of seminal fluid stored after insemination by either one male or two males. The surface area was estimated using the formula π ×1/2a×1/2b, where *a* and *b* were the two axes of the oval capsule (Figure 1A). Female wing length was taken as a proxy measure of body size for the calculation of relative BI surface area. Digital pictures and measurements were taken using ‘analySIS B’ software. Mating duration was recorded for both first and second copulations.

### Experiment 4. Fertility of females when mated twice

The effect of the time between two copulations on the use of sperm for egg fertilization was studied; a second mating opportunity was offered to a mated female, either “immediately after mating” (less than 40 min after the first one, before the BI content gets dense), “after 3 h” (when the BI content was dense), “after 48 h” (when the BI content had dissolved) or “after oviposition” (to test the effect of egg laying on female mating behavior). For each treatment, half the females were mated first to an untreated male and second to a sterilized male (U-S mating sequence); the other half of females was mated to two males in reverse order (S-U). Only females that copulated uninterrupted for more than 30 seconds in a first mating were offered a second mating opportunity. Each female was kept in a separate tube (diameter 2.5 cm) to assess individual fertility over several GC. Tubes were covered with thin netting, which allowed females to take a blood meal daily on the arm of a human volunteer. The bottom of the tube was lined with crepe paper and a small amount of deionized water to moisten the paper for oviposition. After each oviposition, the female was transferred into a new tube and the eggs were matured before hatching. The egg hatch rate was recorded for each GC. In previous studies on *Anopheline* mosquitoes, hatch data gave comparable results as isotopic labeling to reliably indicate the occurrence of multiple mating [43], [44]; an intermediate fertility value indicated the use of sperm from both untreated and sterilized males for egg fertilization. After death, the female was dissected, the number of full spermathecal capsules was recorded and female and male wing sizes were measured. Each interval treatment was replicated between 10 and 18 times with untreated and sterile males in each combination, using different cohorts. When the number of eggs oviposited by a female remained below 30, oviposition was considered incomplete and the fertility value was not taken into account for data analysis.

### Experiment 5. Male insemination ability across several mating sequences

Twenty-three untreated males and 25 sterilized males were used to estimate the number of females that a male can fertilize during its adult life. Four mating periods were selected separated by resting periods of three days. During a first mating period, each 48-hour-old male was proposed 10 virgin females in rapid succession. During this succession, the male was proposed one female at a time; the mated female was removed after copulation and the next female was immediately added. During the first period, the male could mate with a maximum of 10 females. Males were then allowed to rest in the emergence tube for three days between subsequent mating periods; a cotton ball dipped into a 10% sugar solution was available. For the second, third and fourth mating periods, males (then 5, 8, and 11 days old respectively) were provided with a maximum of five virgin females in rapid succession, in the same way as the initial 10 matings took place. If no mating occurred for one hour after addition of a female the test was stopped and the male was once again isolated and allowed to rest. Each mated female was isolated in a tube and frozen 1 hour after the copulation; the BI and spermathecae were dissected, and the surface of the BI was measured if well enough preserved. Female and male wing sizes were measured.

### Ethics Statement

The colony of *Ae. albopictus* was imported in 2010 from the Centro Agricultura Ambiente, Bologna, Italy, in accordance with the Veterinärbehördliche Einfuhrverordnung 2008 – VEVO 2008 of the Federal Ministry of Health of Austria. Research carried out on invertebrates such as mosquitoes do not require a specific permit according to the directive 2010/63/EU of the European Parliament and of the Council on the protection of animals used for scientific purposes. The experiments were performed at the IPCL in Seibersdorf, Austria, respecting the Standard Operating Procedure in force at the laboratory concerning mosquitoes. The blood used for routine blood-feeding was collected in Bratislava and purchased from the Slovak Academy of Sciences. The blood is collected during routine slaughtering of cows in a national abattoir of the highest possible standards that follows strict EU laws and regulations. The occasional blood-feeding of mosquitoes on human forearm (CFO/DD) was performed in accordance with the IPCL rules and following medical control.

### Data analysis

All statistical analyses were carried out using R software [45]. For all the tests, the alpha level was *P*<0.05. Shapiro and Bartlett tests were performed to test the normality and the homoscedasticity of the data, respectively.

The distribution and the average duration of copulation events were compared between untreated and sterile males using a two-sample Kolmogorov-Smirnov test and a two-tailed paired Student's t-test, respectively. The effect of sterilisation or copulation duration on the insemination success was tested using binary logistic regressions. The proportion of successful inseminations for each class of copulation duration and the proportion of females with 1, 2 or 3 filled spermathecae were compared between untreated and sterile males using a proportion test with Yates correction and a Pearson's chi-squared test, respectively. Logistic regressions with a three level categorical variables were used to test the effect of male irradiation, copulation duration, and the number of previous matings on the number of full spermathecae. Differences between the duration of first and second copulations were tested using a two-tailed paired Student's t-test. Proportions of copulations lasting less than 30 seconds were compared between copulation of once-mated females and second copulation of double-mated females using proportion tests with continuity correction. BI surface measurements were square-root transformed to reach normality and homoscedasticity, prior to performing a one-way ANOVA to test the effect of the female mating status. For the double-mating experiment, the effects of the GC, male treatment and the interaction of both, on female fertility and fecundity were tested using repeated-measures two-way ANOVAs. For the male rapid succession mating experiment, the effects of number of previous matings by the male (i.e. mating history), male treatment and the interaction of both on copulation duration were tested using repeated-measures two-way ANOVAs; their effects on copulation success (n observation = 775), insemination success (n = 557), and spermathecal fill (n = 556) were tested using a generalized linear mixed model. The success of insemination for the first three females of each of the three remating periods was compared between untreated and sterile males using a logistic regression. Proportions of females with 1, 2 or 3 spermathecae filled were compared between untreated and sterile males using a Pearson's chi-squared test.

Values in the text are expressed as mean ± SEM.

## Results

### Experiment 1. Copulation of virgin males and insemination success

**Figure 2:**
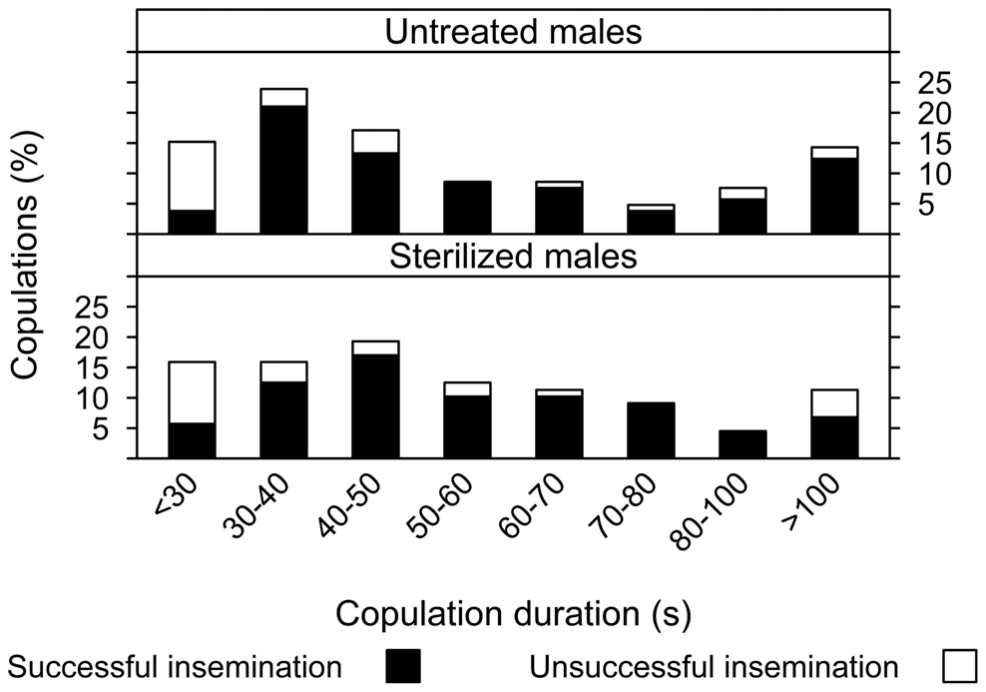
Copulation of virgin male *Ae. albopictus* and insemination success. Percentage of copulations with untreated or sterile males leading to successful (black bars) or unsuccessful (white bars) insemination, in relation to copulation duration.

Copulation duration of untreated males varied from 11 to 338 s with one third lasting from 30 to 50 s (Fig. 2). Sterilized males showed a similar frequency distribution of copulation duration (two-sample Kolmogorov-Smirnov test, D = 0.075, *P* = 0.95). The mean mating duration was not significantly different between untreated and sterilized males (two-tailed paired Student's t-test, t = 0.603, df  = 191, *P* = 0.547); median values indicated a similar value of ca. 45 s (Table 1). Overall, ca. 80% of the copulations were successful (i.e. resulted in insemination) for both sterilized and untreated males, and there was no effect of the irradiation treatment (binary logistic regression, z = −0.18, df  = 182, *P* = 0.86). Figure 2 shows the durations of copulation, separated into classes of 10 s, except when the number of observations was lower than 10. For a given duration class, the proportion of matings which resulted in insemination did not differ significantly between untreated and sterilized males (proportion test with Yates correction, X^2^ = 6.67, df  = 9, *P* = 0.67). However, the probability of a successful insemination was significantly affected by the duration of copulation (binary logistic regression: z = 2.17, df  = 182, *P*<0.05); both short (<30 s) and very long (>100 s) copulations were less successful. For untreated and sterilized males, respectively, 9.5 and 11.4% of all the copulations observed lasted ≤20 s and only 10% of these matings resulted in insemination (Fig. 2). On the other hand, copulations lasting between 30 and 100 s were successful in 91.3±3.4% and 93.3±3.3% of the cases for untreated and sterilized males, respectively. Female size, as indicated by wing size, did not significantly differ (one-way ANOVA, F_(1,77)_  = 1.13, *P* = 0.29) across the groups.

**Table 1:** Duration of copulation, insemination success and spermatheca fill for untreated and sterilized male *Ae. albopictus*.

	Copulation duration (s)			Spermathecae fill per copulation as proportion of total (%):		
Male	Mean ± sem	Median	Successful insemination (%)	1 capsule	2 capsules	3 capsules
Untreated	61±4.7	44	80.8	5	91.3	3.8
Sterilized	56.3±3.9	47.5	79.8	4.5	91	4.5

105 and 88 copulations were observed for untreated and sterilized males, respectively. There was no significant difference between untreated and sterile male values for any of these parameters (*P*>0.05).

Of all the successful matings, the proportions of females with 1, 2, or 3 filled spermathecae (Table 1) did not differ between untreated and sterilized males (Pearson's chi-squared test, X^2^ = 0.0998, df  = 3, *P* = 0.99). There was no effect of male treatment (logistic regression with three-level variable, z = 5.74, df  = 146, *P* = 0.88) or copulation duration (z = 4.32, df  = 146, *P* = 1) on the number of spermathecal capsules filled. Less than 5% of females had all 3 spermathecal capsules filled, and in half of these females only a small portion of the third capsule was observed to be filled with sperm.

### Experiment 2. Sperm transfer dynamics in the female reproductive tract

**Figure 3:**
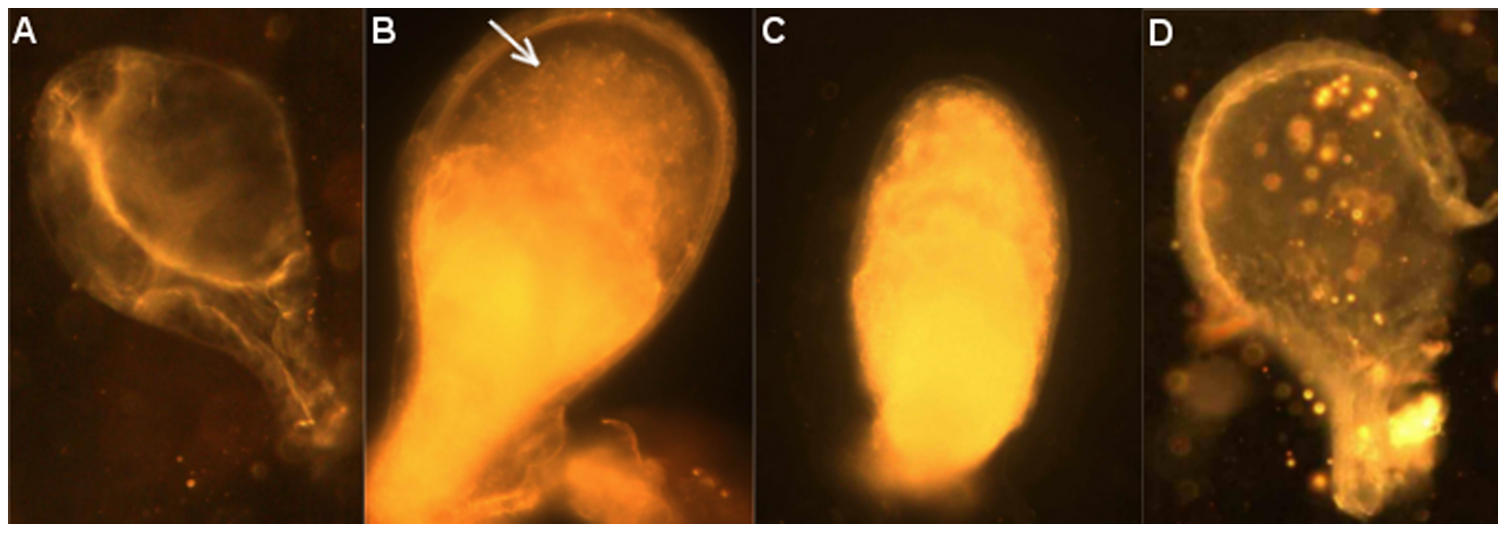
Photographs of the bursa inseminalis (BI) of a virgin female *Ae. albopictus* (A), and inseminated females dissected after 5 min (B), 1 hour (C), and 48 h following insemination (D). The arrow indicates sperm cells still motile in the BI.

After copulation, the transferred sperm was first stored in the empty BI (Fig. 3A & B). The transfer of sperm cells from the BI to the spermathecae was initiated 2 to 3 minutes after the start of copulation, and was terminated for all females 6 minutes after the start of copulation. Inside the BI, sperm cells could be visually identified from the granular mass of the MAG secretions by their shape at a 200 X magnification. We observed that a large quantity of sperm cells remained trapped in the bursa of all females and was not transferred to any spermathecae (Fig. 3C). The granular mass in BI of females that were dissected 15 to 30 min after the copulation appeared denser and the movement of the sperm cells was sparser. The solidification appeared to start at the base of the BI, close to the junction with the common oviduct. The BI content appeared completely dense in all females dissected 40 min to 6 h after copulation. The beginning of the dissolution of the BI content was not observed, but 80% of the females had an empty BI after 24 h, and depletion was complete in all the females dissected 48 h after copulation. After dissolution of the BI contents, a very small quantity of remnant material could be observed in the BI (Fig. 3D).

### Experiment 3. Transfer of semen in once- or twice-mated females

No difference was found between the mean duration of the second copulation of twice-mated females as compared to that of once-mated females (two-tailed paired Student's t-test, t = 0.307, df  = 81.1, *P* = 0.76). However, the proportion of copulations lasting less than 30 s differed significantly (48.5% for second copulation of twice-mated females, and 4.5% for once-mated females; proportion test with continuity correction: X^2^ = 31, df  = 1, *P*<0.001).

**Figure 4:**
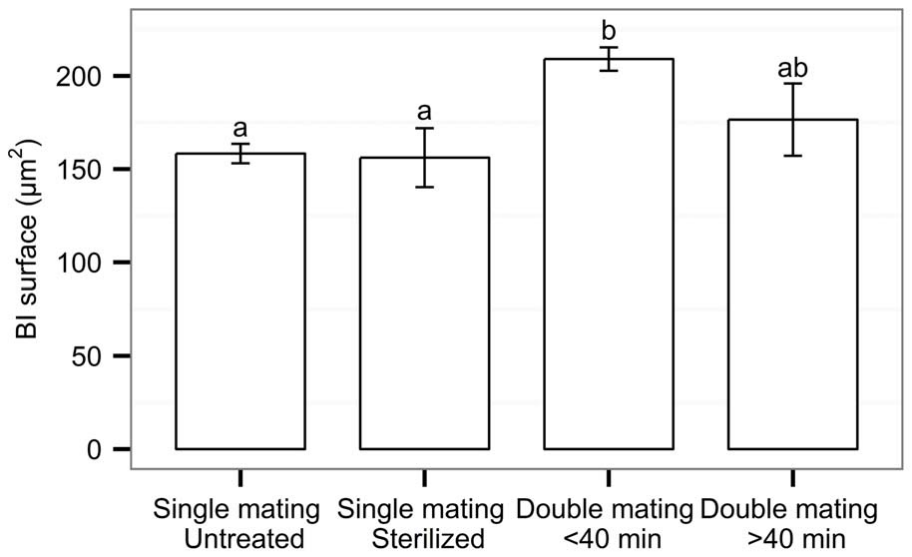
Sperm transfer to the *Ae. albopictus* female bursa inseminalis (BI): mean (+ SEM) relative BI surface area according to the mating status of the female. N was 61, 15, 57 and 9, respectively, for females inseminated once by an untreated male or a sterile male, and females inseminated twice in an interval shorter or longer than 40: ANOVA, *P*<0.05.

The number of matings significantly affected the relative BI surface area in females (two-tailed paired Student's t-test, t = −5.78, df  = 118, *P*<0.001; Fig. 4). The relative BI surface area of once-mated females with either an untreated or a sterilized male was on average significantly smaller as compared to twice-mated females, when the interval between the two matings was less than 40 min (One way ANOVA, F_4,120_ = 9.93, *P*<0.001; Tukey Post-Hoc tests, *P*<0.001 for both untreated and sterile mates). Around 11% of the females mated twice in an interval of less than 40 minutes showed a larger BI surface area than the maximum surface observed in once-mated females. In two of the twice-mated females two distinct bodies of semen were visible in the BI (Fig. 1). When the second mating occurred more than 40 minutes after the first one, the relative BI surface area was not significantly different from that of females mated once or females that were mated twice in an interval of less than 40 minutes. The number of matings did not affect the number of filled spermathecal capsules in the females (logistic regression, z = 1.48, df  = 126, *P* = 0.52).

### Experiment 4. Fertility of females when mated twice

**Figure 5:**
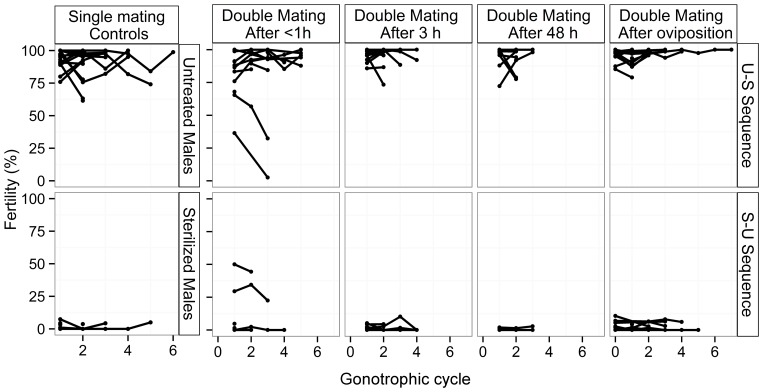
Fertility of female *Ae. albopictus* mated once with an untreated or sterilized male, or twice at various interval of time with males in untreated-sterilized or sterilized-untreated mating sequences. Individual fertility of females over multiple gonotrophic cycles.

Across all mating treatments female size, as indicated by wing size, did not significantly differ (one-way ANOVA, F_(7,22)_  = 1.5, *P* = 0.22). Figure 5 represents the variation of individual females' fertility over the GCs one to six, under each mating treatment and according to the mating sequence. In the single mating treatment, the fertility of each female mated by an untreated or a sterilized male was on average 94,4±0,8 and 1.2±0.2%, respectively, for the first GC, and remained similar for all the following GCs (repeated-measures ANOVA, untreated mate controls: F_(1,27)_  = 1.14, *P* = 0.3; sterilized mate controls: F_(1,7)_  = 0.49, *P* = 0.51).

In the double-mating tests, twice-mated females of both the U-S and S-U mating sequence showed a similar fertility to their respective controls except in the situation where the second mating occurred immediately (less than 40 min) after the first one (one-way ANOVA, U-S, F_(4,164)_  = 4.07, *P*<0.01; S-U, F_(4,105)_  = 9.21, *P*<0.001). In this treatment, 15% of the females showed an intermediate fertility value during the first GC. The intervals separating the two copulations of twice-inseminated females ranged from 4 to 35 minutes. In the U-S sequence, the fertility of the twice-inseminated females decreased during the second and subsequent GCs (repeated-measures ANOVA, F_(1,4)_  = 36.4, *P*<0.01), whereas the fertility of females from the S-U sequence remained stable across the subsequent GCs (F_(1,2)_  = 1.57, *P* = 0.34). All other twice-mated females showed either high fertility, or high sterility which remained stable during all the GCs (repeated-measures ANOVA, F_(1,289)_  = 3.65, *P* = 0.057).

The mean fecundity for the first GC was 64±5 eggs per female, and slightly decreased to a mean of 51±7 eggs at the 5^th^ GC. Fecundity varied significantly over the GCs (repeated-measures ANOVA, F_(1,301)_  = 873, *P*<0.01), except during the first two (F_(1,219)_  = 3.62, *P* = 0.058). Fecundity was independent from the father (s) treatment status (F_(4,298)_  = 2.04, *P* = 0.088) and from the female body size (F_(1,28)_  = 0.02, *P* = 0.89). We observed that sperm was still present in two of the spermathecae for all the females dissected after undertaking 6 GCs.

The mean duration of the second copulation was significantly shorter when it occurred 3 h after the first one compared to immediately after (One way ANOVA, F(_3,253_ = 3.63, *P*<0.05; Tukey Post-Hoc tests, *P* = 0.022); but it was significantly longer when it occurred 48 h after the first copulation as compared to 3 h after (Tukey Post-Hoc tests, *P* = 0.038). The proportion of copulations lasting less than 30 s was significantly higher when the second copulation occurred 3 h after the first mating or after oviposition (proportion test, X^2^ = 19, df  = 3, *P*<0.05; Pair-wise comparison of proportions, *P* = 0.003) as compared to second copulations occurring within 40 min after the first mating.

### Experiment 5. Male insemination ability across several mating sequences

Across the two treatments, male and female size did not differ significantly. The first sequence of 10 copulations in rapid succession lasted from 1 to 6 h for a single male, with a mean of 3.6±1.5 h for both untreated and sterilized males. The time separating two successive copulations ranged from 0 to 2 h with an average of 15 min for both male treatments. Copulation duration was significantly affected by the male treatment (repeated-measures ANOVA, F_(1,544)_  = 5.00, P<0.05) but not by the number of previous matings across all mating periods (F_(1,544)_  = 1.72, *P* = 0.191).

**Figure 6:**
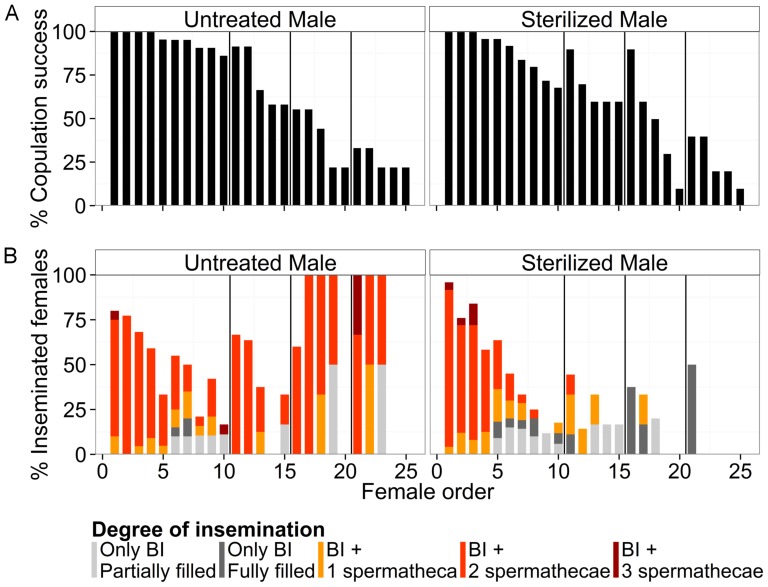
Insemination capacity of a male *Ae. albopictus* mated with several females in rapid succession. Percentage of copulation success on all trials (A) and percentage of insemination success of all copulations (B) according to order of mating opportunities, for untreated and sterilized males. Various degrees of female insemination are represented by different coloration within the bars. The vertical lines within the graphs divide the four mating periods.

Refusal (i.e. no attempt) or failure (i.e. unsuccessful attempts) of males to copulate increased with the number of previous matings (Fig. 6A). Male copulation success (regardless of insemination success) was significantly affected by irradiation (generalized linear mixed model, log likelihood  = −230, z = 2.0, *P*<0.05) and number of previous matings across all mating periods (z = −12.44, *P*<0.001).

Untreated males were able to inseminate females during each mating period, although a cyclic pattern of decreasing and increasing insemination success was observed over the successive copulation attempts (Fig. 6B). The insemination success (i.e. copulation with transfer of semen to the female) of males was significantly affected by male treatment (generalized linear mixed model, log likelihood  = −351, z = 2.99, *P*<0.01), number of previous matings across all mating periods (z = −6.4, *P*<0.001), and the interaction of both (z = −4.42, *P*<0.001). During the first mating period, the insemination success of a 2-day-old untreated male decreased from an averaged 80±9.2% for the first female offered to 16.7±9% for the 10^th^ copulation.

Among the inseminated females, the number of filled spermathecae and the filling of the BI varied greatly over the series of copulations (Fig. 6B). This degree of insemination was significantly affected by male treatment (generalized linear mixed model, log likelihood  = −303, z = −4.86, *P*<0.001), number of previous matings across all mating periods (z = −2.37, *P*<0.05), and the interaction of both (z = −6.46, *P*<0.001). During all mating periods untreated and sterilized males were able to transfer sperm to at least one spermatheca of a maximum of 11 and 7 females, respectively, and to transfer semen to the BI (but not spermathecae) of an additional maximum of 9 and 8 females, respectively. More than 80% of the first five females inseminated by an untreated male during the first mating period had two spermathecae filled with sperm. The proportions of females with 0, 1 or 2 spermathecae filled with semen were not significantly different between females mated with sterilized and untreated males over the first mating period (Pearson's Chi-squared test, X^2^ = 1.6, df  = 3, *P* = 0.66). However, during the following mating periods the number of spermathecae filled in each mating continued to decrease, and the proportions differed significantly between females that mated with sterilized and untreated males (X^2^ = 38.8, df  = 3, *P*<0.001). Fifty, 83 and 100% of the females inseminated by a sterilized male had semen only in the BI during the second, third and fourth mating periods, respectively. In some instances where only the BI was filled with semen, observations under the microscope indicated the presence of sperm cells but little granular mass in the BI.

## Discussion

This study brings a better understanding of the mechanisms of sperm transfer in various situations and on the likelihood of multiple insemination of female *Ae. albopictus,* and gives an indication of the sexual strategy of the male sex of the species. However, colonized *Ae. albopictus* mosquitoes were used for these laboratory-based investigations, and it would be interesting to verify the behavior of male and female in response to a first mating with wild mosquitoes.

We showed that one of the most important events during the sperm transfer and storage in the female reproductive tract was the storage and solidification of the mixture of MAG secretion and sperm cells within the BI. This content appeared to play a crucial role in controlling a female's likelihood to be multiply-inseminated. In case of double-copulation, the female had a greater chance to be inseminated by both males only if both copulations occurred within a 40 min interval. Longer intervals of time resulted in the birth of offspring fathered only by the first male, over several gonotrophic cycles. When several females were offered to males in a rapid sequence of copulations, most males attempted to copulate irrespective of the available sperm supply or accessory gland secretion material. The quantity of sperm transferred by males over a sequence of copulations decreased, as shown by the number of spermathecae filled with sperm. Untreated males were able to recover their full insemination ability after a few days of rest without copulation, whereas sterile males were not.

### Sperm transfer in female *Ae. albopictus* and the role of the bursa inseminalis content in preventing multiple insemination

This study has shown that *Ae. albopictus* males transferred a large amount of MAG secretions together with motile sperm during mating; this package is stored in the BI before migration of the sperm cells to the spermathecal capsules. The transfer of sperm did not occur immediately after coupling, as was evidenced by the absence of sperm transfer during most of the copulations that lasted less than 30 s. This suggests that the first moments of the copulation act could be devoted to pre-insemination actions, probably concentration of sperm and MAG material in the male reproductive organs or getting a productive physical interlock. A similar mechanism of voluminous material transfer has been observed previously for *Ae. aegypti* [46], but such a pre-insemination period does not appear to be necessary for this species since Spielman et al. [22] reported that the transfer of semen from the male to the female BI occurred after only 4 s of contact, and successful insemination occurred after 6 s. The whole duration of copulation was also considerably longer for our *Ae. albopictus* strain (on average 45 s in emergence tubes under free mating conditions) as compared to *Ae. aegypti* which averaged 13 s in a lantern chimney [46] and 16 s in larger cages [33]. Ponlawat and Harrington [47] reported that wild males copulated for a shorter time than males from laboratory-reared strains; further studies on the behavioral differences between laboratory and wild strains would be highly valuable.

We observed that the migration of sperm from the BI to the spermathecae was completed within the first 6 minutes after the start of copulation, similarly to *Ae. aegypti* [46]. The kinetics for the BI content to get dense and dissolve in female *Ae. albopictus* were also comparable to *Ae. aegypti* [48]; around 40 minutes to 1 hour after insemination the medium appeared too dense for sperm cells to be able to move about. Observations under a microscope revealed that numerous sperm cells remained trapped within the BI when the granular content solidified completely, and therefore never reached any spermatheca. Jones and Wheeler [46] estimated that only 62% of the sperm transferred by *Ae. aegypti* reached the spermathecae, the remaining cells being trapped in the MAG secretions inside the BI. Despite this apparent excess of sperm transferred by male during the copulation, the third spermatheca was filled with sperm in only ca. 4% of the *Ae. albopictus* females inseminated by virgin males in this study. In this species, as in *Ae. aegypti*, it is still unclear why the transfer of spermatozoids usually stops after the filling of two spermathecae despite large amounts of sperm cells remaining in the BI [24]. This extra sperm is eventually dissolved within a day together with the MAG secretions in the BI. In evolutionary terms, the existence of this apparent waste of sperm might signify that it implies very low costs for the male or/and that the extra sperm might be important to establish the physical barrier that prevents a further insemination of the female, thus ensuring paternity of the progeny. This excess of sperm might as well have some nutritious value for the female, resulting in a better survival or fecundity, therefore impacting on the male's fitness. Another possible hypothesis would be that the female does not store all the transferred sperm thus keeping space for a potential further insemination by different males and allowing for sperm competition.

The dense BI content seems to act as a physical barrier preventing a second insemination in female *Ae. albopictus* when the successive mating event takes place more than 40 minutes after the first one. In addition, we reported a high proportion of short second copulations (lasting less than 30 s) when they took place 40 min or 3 h after the first mating, suggesting a premature termination of the copulation by the second male probably due to the presence of a solid mass in the female's BI or a rejection by the female. Male *Ae. aegypti* have also been observed to prematurely terminate copulation when mating with non-virgin females [49]. On the other hand, when the period separating two matings was shorter than 40 minutes, we observed visual signs of a second semen transfer as suggested by a larger relative BI surface area in ca. 11% of twice-mated females and by an intermediate fertility values in ca. 15% of females. Craig [50] likewise reported that a second successful insemination might be possible for *Ae. aegypti* if mating occurred before the BI content was getting dense.

However, the physical barrier created by a dense mass in the BI is not the only mechanism that would prevent a second male from siring offspring. When sufficient time was left between two copulations for the BI content to dissolve, the second male *Ae. albopictus* was still not able to transfer sperm into the female's storage organs as indicated by the uniformity of the either fully fertile or sterile progeny oviposited over several GCs. A second insemination was still not effective even after a blood-meal and oviposition. A similar situation was reported in *An. gambiae* females over five GCs [51]. These outcomes corroborate the findings about the MAG in mosquitoes [52] and more particularly in aedines [31], where MAG products were shown to prevent the insemination of *Ae. albopictus* and *Ae. aegypti* females. In their study, Helinski et al [31] did not give details about the copulatory behavior of females; however, we observed no apparent decrease of female receptivity since mated females did not refuse copulation with successive males. This suggests that monogamy in *Ae. albopictus* would not be ruled by the female's behavior. Rather than an inhibition of female sexual receptivity, we suggest that it is the sperm transfer to the spermathecae that is inhibited by the first insemination. It therefore appears that, similarly to tephiritids [53], [54] the possibility of re-insemination of *Ae. albopictus* females is physically inhibited in the short term by the dense granular mass inside the BI, and by a longer-term biochemical effect that is later induced possibly by the MAG secretion. It is yet unknown how the MAG secretions act to hinder a female's re-insemination, although the nature of MAG secretions [32], [55] and their transcriptional regulation [56] are being unraveled. Considering the outcomes of our study and the recent report of multiple-inseminated female *Ae. albopictus* encountered in the field [36], we can assume that wild virgin females are subjected to several mating attempts by different males in a short period of time and probably soon after emergence or during their first blood-meal.

When offered a mated female, males usually attempted to copulate even though these matings had a low probability of fertilizing embryos. However, the average shorter duration of the female's second copulation suggests that the second male might be able to detect his mate's mating status during the copulation, before any or after a partial transfer of sperm and MAG. If a partial transfer and storage of sperm occurs, then the reproductive success of the male increases. In mosquitoes, sperm competition has been poorly studied but it appears that this phenomenon would be limited to matings that are closely spaced in time. Our results suggest that when sperm from two different males are present in a female spermathecae, both participate to the fertilization of the eggs on an equal basis during the first oviposition. Indeed, the fertility of twice-inseminated female *Ae. albopictus* ranged from 30 to 65% in the first GC. Over successive GCs, an individual female's fertility decreased or remained stable, but no common pattern could be observed. We can hypothesize that variation in fertility levels depended on the respective amounts of sperm transferred by each male, which might vary with the interval between the two mating events.

### *Ae. albopictus* males attempt to copulate irrespective of their sperm supply

In the rapid sequence mating experiment, male *Ae. albopictus* usually copulated with all 10 females they were offered on the first day of test, but fully inseminated only the first five. Gamete management has evolved in male insects in response to factors such as the number, quality and spatial and temporal dispersion of the reproductive opportunities that adults encounter [11]. In *Ae. albopictus*, females (mating opportunities) appear to be aggregated spatially and temporally around blood meal sources or breeding sites. The ability of males to parcel ejaculate in order to successively inseminate at least five females during one day would enable them to take advantage of such high probabilities to encounter females in order to increase their fitness.

Males *Ae. albopictus* showed a propensity to mate with most females encountered, which could benefit them by transferring sperm in virgin and recently mated females. The cost of this behavior could be lowered by limiting the copulation duration with a female that has mated long time before. The high probability of encountering females around the blood-feeding sources has probably lead to the selection of this behavior. The amount of semen transferred to most of the last-ranked females decreased progressively, resulting in only one spermatheca filled or none at all in the end, even though the BI was filled. A similar phenomenon was reported for *Ae. aegypti* in rapid sequence matings [34], [57]. Some studies reported the complete depletion of sperm from the seminal vesicles, *vas deferens* and *vas efferens*, and depletion of secretory material from the accessory glands in *Ae. aegypti* after five successive inseminations [34], [58]–[60]. Lum [48] showed that successive matings of a male resulted in the exhaustion of the MAG contents before the vesicles were exhausted of spermatozoa. Our observations of last-ranked females corroborate Lum's statement as we observed that some sperm cells but very little MAG secretion were present in the BI. In those females no sperm was transferred to the spermathecae; the MAG secretion is believed to serve as a transport medium for the spermatozoa [48], therefore a lack of secretion might make migration of spermatozoa impossible. In *Ae. aegypti* and *Ae. albopictus* females, an implant of MAG was sufficient to provide an oviposition stimulus [61], and injection of a low dose of MAG secretion (from 4.2% of one male secretion) could prevent reinsemination of a female [31]. It is therefore possible that these incomplete inseminations when few sperm and MAG have been transferred may still diminish a female's propensity to remate, increasing the relative fitness of the male. Such a phenomenon has been suggested in the case of copulation of sperm-depleted hymenoptera males [62].

Male *Ae. albopictus* showed some characteristics of parcimonious sperm allocation [6] as they are able to parcel ejaculated sperm over a series of matings, and seem to adapt the duration of the copulation according to the female mating status. On the other hand, a large amount of transferred sperm never reaches any spermathecae, and males attempt to copulate regardless of their recent mating history (level of sperm or MAG depletion), which appear contradictory with a parsimonious sperm allocation. The high quantity of sperm and MAG transfer has probably evolved under a high male competition situation, leading to mechanisms of avoiding sperm competition that prevent mated females to store sperm from another male. Further investigations on the ability of male *Ae. albopictus* to detect the female mating status and regulate the duration of copulation and the quantity of sperm transferred would bring precious information to understand their mating strategies.

### Impact of *Ae. albopictus* male's reproductive behavior on SIT programs

The outcomes of this study provide as well some insights with respect to the SIT as a vector control method. Copulations with sterile males did not differ from those by untreated males in duration or approximate amount of sperm transferred, as estimated from the female BI surface.

After a resting period without sexual activity, untreated male *Ae. albopictus* were once again able to fully inseminate females, which is consistent with the observations of new mature sperm cells in the testes and replenishment of MAG secretory material after depletion in some mosquito species, including *Ae. aegypti* [58], [63]–[65]. However, sterile males were not able to replenish their sperm stock once depleted. Over its lifetime, one sterile male might fully inseminate (fill at least one spermatheca) a maximum of 7 females, and might transfer a partial amount of semen (filling only the BI) to a further maximum of 8 females. Over the same period of time, untreated males could fully inseminate up to 11 females and partially inseminate another 9 females. Similar outcomes have been reported for the Reunion strain of *Ae. albopictus* during the first 9 days of matings; the subsequent reduction in mating ability observed [66] was therefore likely due to a depletion of semen in sterile males. In adult males, new spermatozoa are released from mature spermatocysts into the seminal vesicles [47]; the replenishment of the sperm supply would not occur as it is transferred during matings, but would require the formation and maturation of about 11 extra cysts [67]. A reduction of the total number of females inseminated by a sterile male was thus expected, as the irradiation damages are higher in the earlier stages of spermatogenesis (spermatogonia and spermatocytes) than in mature sperm cells, and therefore impede the immature spermatocytes from developing further [68]. For other insect species, a reduction of sperm quantity or quality [69]–[72] and a faster emptying of testis have been reported [73], [74] with the sterilization process.

In the context of SIT it is critical to understand how sperm dynamics of irradiated males can influence female reproductive and remating behavior, and determine whether multiple mating, caused by unequal insemination ability (quality and quantity of sperm and MAG products transferred) in sterile and wild males, could decrease the efficiency of SIT [75]. In the current study, sterile male *Ae. albopictus* proved able to transfer enough semen to the females to prevent a further insemination, similarly to untreated males. Therefore in a program with an SIT component, released sterile males could be expected to compete equally with wild ones, providing that they survive well and are able to locate the wild females under the natural conditions. However, these outcomes highlight the necessity of identifying the mating sites for this species in order to optimize the sterile males release strategy, if the releases are not aerial.
